# Supplementary figures and images for: Chorioamnionitis as a risk factor for retinopathy of prematurity: An updated systematic review and meta-analysis
Source: PLoS One. 2018 Oct 17;13(10):e0205838. doi: 10.1371/journal.pone.0205838 (PMC6192636; doi:10.1371/journal.pone.0205838)

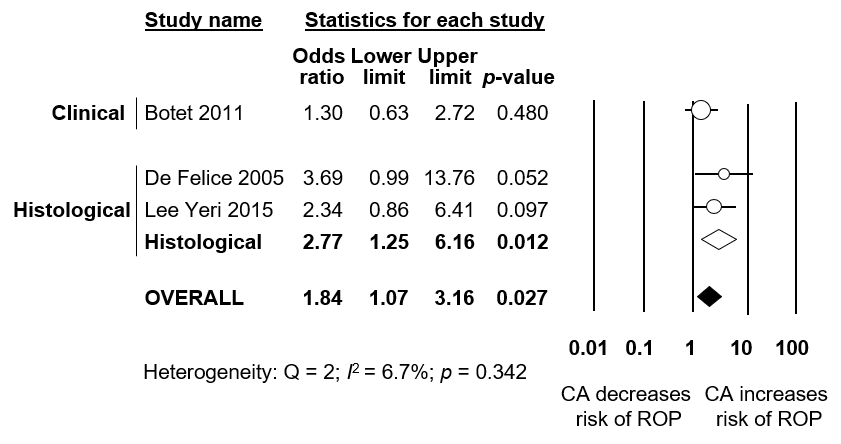

Supplement: S1 Fig — CA: chorioamnionitis; ROP: retinopathy of prematurity. (TIF) [file pone.0205838.s001.tif]

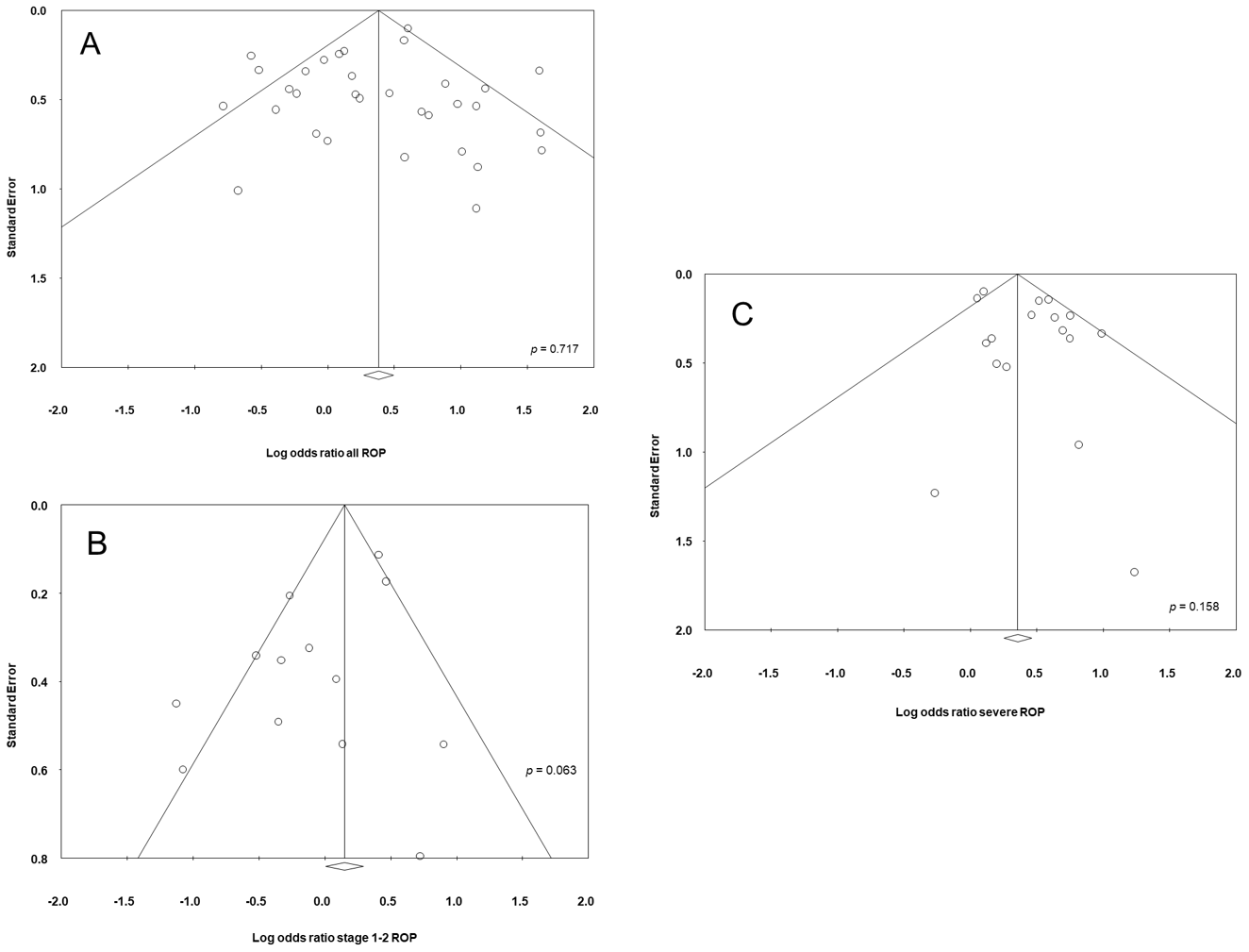

Supplement: S2 Fig — CA: chorioamnionitis; ROP: retinopathy of prematurity. (TIF) [file pone.0205838.s002.tif]

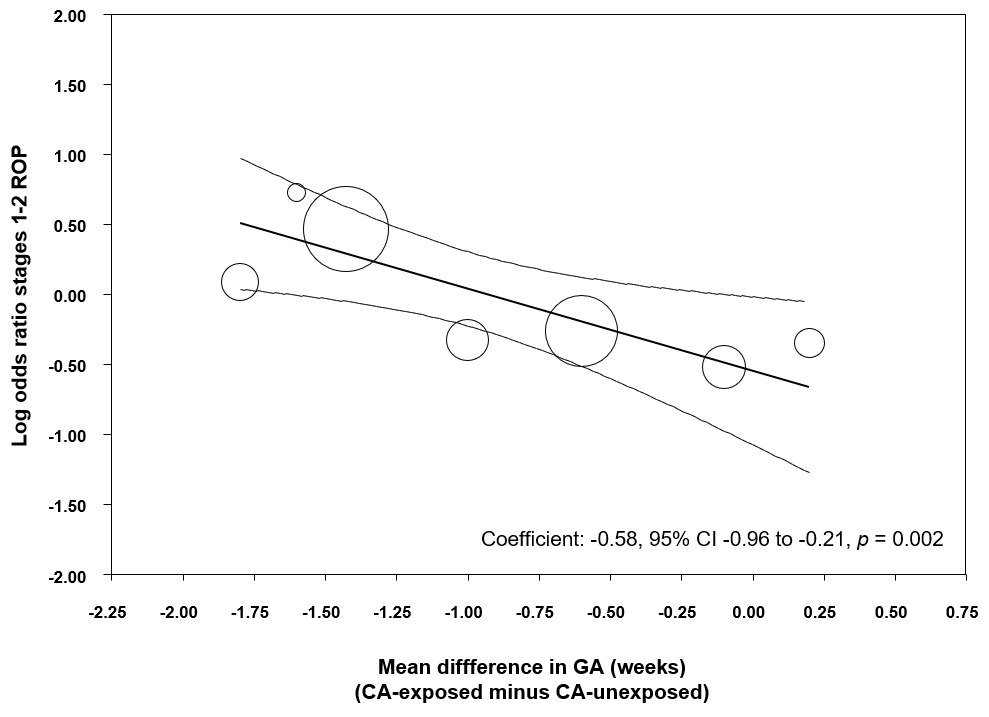

Supplement: S3 Fig — CA: chorioamnionitis; ROP: retinopathy of prematurity; GA: gestational age. (TIF) [file pone.0205838.s003.tif]

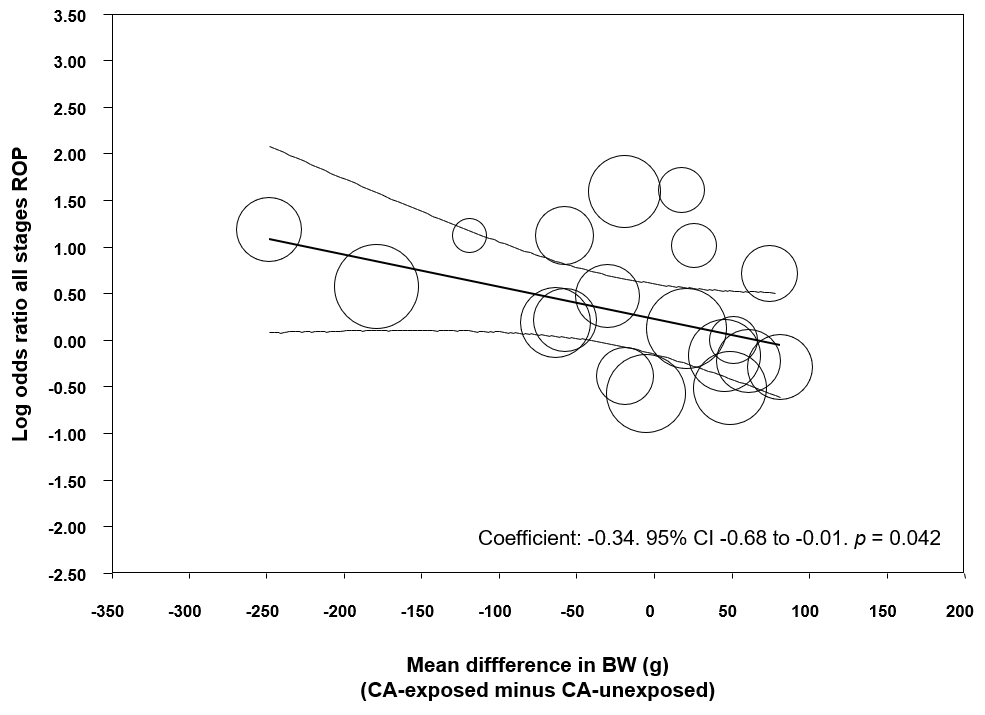

Supplement: S4 Fig — CA: chorioamnionitis; ROP: retinopathy of prematurity. (TIF) [file pone.0205838.s004.tif]

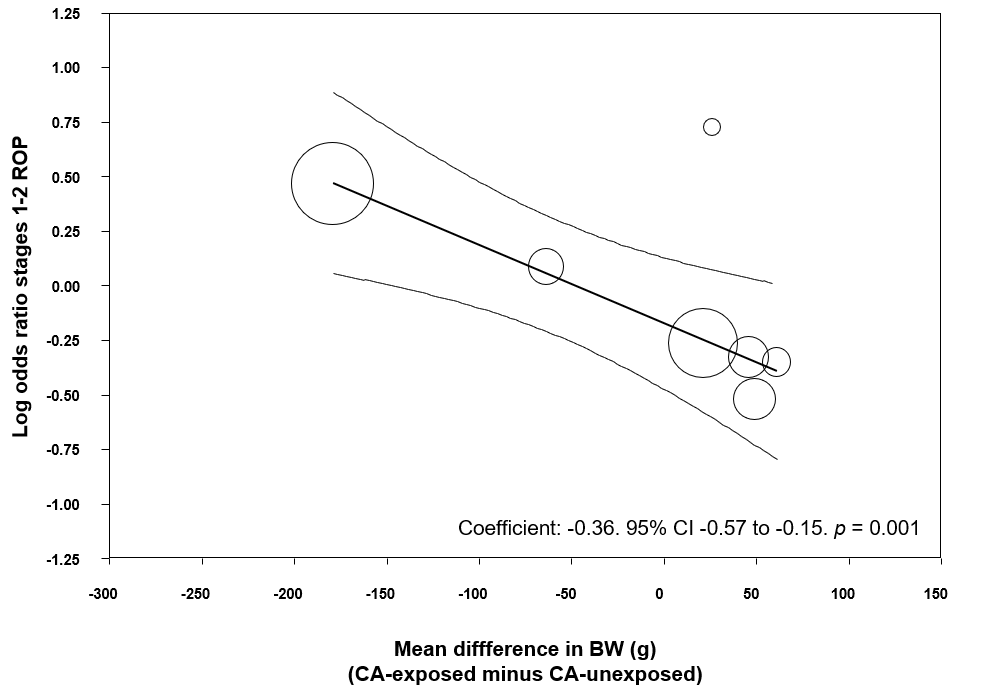

Supplement: S5 Fig — CA: chorioamnionitis; BW: birth weight; ROP: retinopathy of prematurity. (TIF) [file pone.0205838.s005.tif]

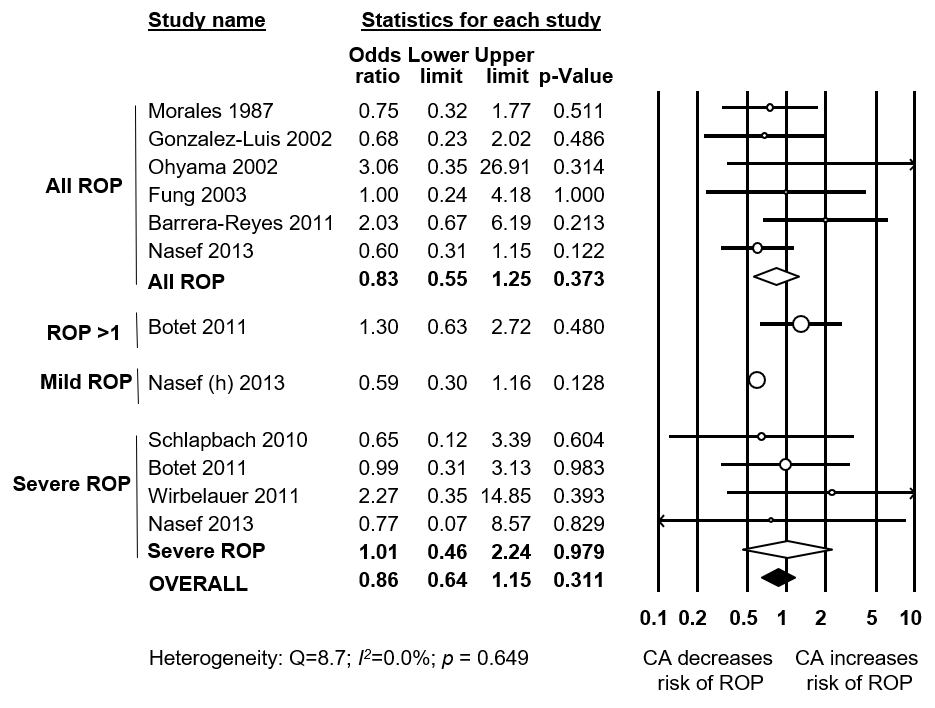

Supplement: S6 Fig — GA: gestational age; CA: chorioamnionitis; ROP: retinopathy of prematurity. (TIF) [file pone.0205838.s006.tif]

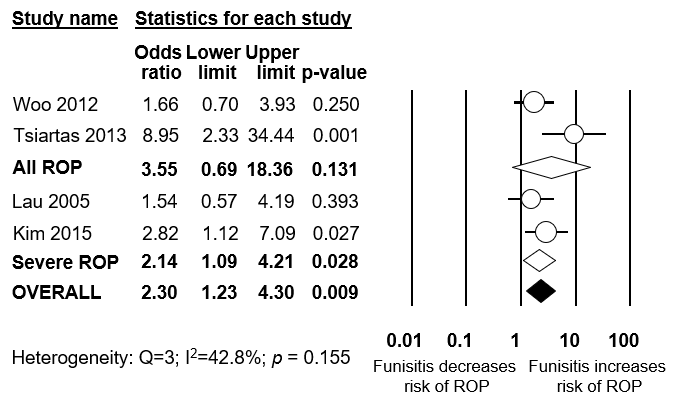

Supplement: S7 Fig — CA: chorioamnionitis; ROP: retinopathy of prematurity. (TIF) [file pone.0205838.s007.tif]
