# Supplementary material for: Chorioamnionitis as a risk factor for retinopathy of prematurity: An updated systematic review and meta-analysis
Source: PLoS One. 2018 Oct 17;13(10):e0205838. doi: 10.1371/journal.pone.0205838 (PMC6192636; doi:10.1371/journal.pone.0205838)
Supplement: S1 Table — (DOCX) [file pone.0205838.s008.docx]

**Table S 1. Search strategy PubMed.**

| (chorioamnionitis OR “intrauterine infection” OR “intrauterine inflammation” OR “antenatal infection” OR “antenatal inflammation”) AND (outcome OR retinopathy of prematurity) AND (cohort OR case control) and (risk factor) |
| --- |
| (retinopathy of prematurity) AND (cohort OR case control) and (risk factor) and (preterm infant OR very low birth weight infant) |
| (chorioamnionitis OR “intrauterine infection” OR “intrauterine inflammation” OR “antenatal infection” OR “antenatal inflammation”) AND (retinopathy of prematurity) |
