# Supplementary material for: Chorioamnionitis as a risk factor for retinopathy of prematurity: An updated systematic review and meta-analysis
Source: PLoS One. 2018 Oct 17;13(10):e0205838. doi: 10.1371/journal.pone.0205838 (PMC6192636; doi:10.1371/journal.pone.0205838)
Supplement: S2 Table — GA: gestational age; BW: birth weight; ACS: antenatal steroids. (DOCX) [file pone.0205838.s009.docx]

**S2 Table. Synoptic table of all included studies.**

| **First author, year** | **City/region, Country** | **Design^a^** | **Perspective** | **Prosp/Retro^a^** | **Total infants (centers)** | **Mean BW (g)** | **Mean GA (weeks)** | **Male (%)** | **ACS (%)** | **CA category^b^** | **Definition of CA^c^** | **Definition of ROP^d^** | **Newcastle-Ottawa Scale Score^e^** | | | |
| --- | --- | --- | --- | --- | --- | --- | --- | --- | --- | --- | --- | --- | --- | --- | --- | --- |
|  |  |  |  |  |  |  |  |  |  |  |  |  | Select. | Comp. | Outc. | Tot. |
| Al-Essa, 2000 | Kuwait City, Kuwait | Cohort | ROP | Prosp | 234 (1) | 1145 | 30,2 | 49 |  | CC | NoDes | ICROP | 3 | 0 | 3 | 6 |
| Allegaert, 2003 | Leuven, Belgium | Ca-Co | ROP | Retro | 62 (1) | 827 |  |  | 58 | HC | NoDes | ICROP | 3 | 0 | 2 | 5 |
| Austeng, 2010 (EXPRESS group) | Sweden | Cohort | CA | Prosp | 497 | 800 | 24,3 | 55 | 71 | CC |  | ICROP | 3 | 2 | 3 | 8 |
| Barrera-Reyes, 2011 | Mexico, Mexico | Cohort | CA | Prosp | 104 (1) | 1071 | 30,0 | 52 |  | CC | Ref | NA | 4 | 0 | 2 | 6 |
| Bordigato, 2010 | Padova, Italy | Cohort | CA | Unclear | 29 (1) | 805 | 26,6 | 59 | 76 | HC | Ref | NA | 4 | 0 | 2 | 6 |
| Borroni, 2013 | Italian ROP study group | Cohort | ROP | Prosp | 421 (25) |  |  | 43 | 79 | HC | NoDes | ICROP | 3 | 0 | 2 | 5 |
| Botet, 2011 | Spain | Ca-Co | CA | Prosp | 328 (12) | 1096 | 28,7 | 54 | 84 | CC | Des | NA | 4 | 0 | 2 | 6 |
| Chen, 2011 | ELGAN study group, USA | Cohort | ROP | Prosp | 1062 (14) |  |  | 54 |  | HCF | Ref | ICROP | 4 | 0 | 3 | 7 |
| Dammann, 2009 | Hannover, Germany | Cohort | ROP | Retro | 73 (1) |  | 28,5 | 48 | 81 | CC | NoDes | NA | 3 | 1 | 2 | 6 |
| De Felice, 2005 | Siena and Brindisi, Italy | Cohort | CA | Prosp | 116 (2) | 977 | 28,1 | 48 |  | HC | Ref | NA | 4 | 0 | 2 | 6 |
| Fung, 2003 | Hong Kong, China | Cohort | CA | Prosp | 72 (1) | 794 | 26,2 | 50 | 83 | CC | Des | NA | 4 | 0 | 2 | 6 |
| Gagliardi, 2014 | Italian Neonatal Network | Cohort | CA | Prosp | 3606 (82) | 938 | 27,4 | 50 | 84 | CC | NoDes | NA | 3 | 2 | 2 | 7 |
| Garcia-Munoz Rodrigo, 2014 | Spanish Network | Cohort | CA | Prosp | 8330 (53) | 1086 | 28,5 | 52 | 67 | CC | Des | NA | 4 | 2 | 2 | 8 |
| Gaugler, 2002 | Strasbourg, France | Cohort | ROP | Retro | 164 (1) | 1007 |  |  |  | CC | NoDes |  | 4 | 0 | 2 | 6 |
| Giapros, 2011 | Ioannina, Greece | Cohort | ROP | Retro | 189 (1) | 1285 | 29,9 | 53 | 58 | CC | NoDes | ICROP | 3 | 0 | 3 | 6 |
| Gonzalez-Luis, 2002 | Barcelona, Spain | Ca-Co | CA | Retro | 135 | 1131 | 28,5 |  |  | CC |  |  | 4 | 0 | 2 | 6 |
| Gray, 1997 | Brisbane, Australia | Cohort | CA | Unclear | 158 (1) | 954 | 27,0 | 56 |  | HC or CC | Des | ICROP | 4 | 0 | 3 | 7 |
| Hendson, 2011 | Edmonton, Canada | Cohort | CA | Prosp | 484 (1) | 930 | 26,9 | 48 | 83 | HCF | Des | ICROP | 4 | 0 | 3 | 7 |
| Holmstrom, 1996 | Stockholm, Sweden | Cohort | ROP | Retro | 202 (5) |  |  |  |  | CC | NoDes | ICROP | 3 | 0 | 3 | 6 |
| Hwang, 2015 | Korean Neonatal Network | Cohort | ROP | Retro | 2009 (55) | 946 | 28,9 | 50 | 76 | HC | NoDes |  | 3 | 0 | 3 | 6 |
| Kavurt, 2014 | Ankara, Turkey | Cohort | ROP | Prosp | 495 (1) | 1267 | 29,3 | 50 | 55 | CC | NoDes |  | 3 | 0 | 3 | 6 |
| Kim, 2015 | Seoul, Korea | Cohort | CA | Retro | 258 (1) | 1104 | 29,2 | 50 | 81 | HCF | Des | ICROP | 4 | 2 | 3 | 9 |
| Lau, 2005 | Canada | Cohort | CA | Prosp | 1296 (1) | 2068 | 33,2 | 55 | 47 | HCF | Ref | ICROP | 4 | 0 | 3 | 7 |
| Lee Hyun Ju, 2011 | Seoul, Korea | Cohort | CA | Retro | 147 (2) | 785 | 26,5 | 56 | 67 | HC | Ref | NA | 4 | 0 | 2 | 6 |
| Lee Yeri, 2015 | Seoul, Korea | Cohort | CA | Retro | 339 (1) | 1525 | 30,0 | 57 | 84 | HC | Ref | ICROP | 4 | 0 | 3 | 7 |
| Liu, 2014 | Chongqing medical university | Cohort | ROP | Retro | 1614 (1) |  |  |  |  | CC | NoDes | ICROP | 4 | 0 | 3 | 7 |
| Martinez-Cruz, 2012 | Mexico, Mexico | Cohort | ROP | Prosp | 139 (1) | 779 |  | 40 |  | CC | NoDes | Ref | 3 | 0 | 3 | 6 |
| Mehta, 2006 | New Brunswick, USA | Cohort | ROP | Retro | 164 (1) |  |  |  |  | HC | Ref | NA | 4 | 0 | 2 | 6 |
| Morales, 1987 | Orlando, USA | Ca-Co | CA | Prosp | 86 (1) | 1178 | 29,2 |  |  | HC & CC combined | Des |  | 4 | 0 | 2 | 6 |
| Mu, 2008 | Taipei, Taiwan | Cohort | CA | Prosp | 119 (1) | 1108 | 28,6 | 54 | 45 | HC | Ref |  | 4 | 1 | 2 | 7 |
| Nasef, 2013 | Toronto, Canada | Cohort | CA | Retro | 179 (1) | 938 | 27,1 | 55 | 85 | HC & CC separately defined | Ref | Ref | 4 | 0 | 3 | 7 |
| Ogunyemi, 2009 | Los Angeles, USA | Cohort | CA | Retro | 774 (1) | 1313 | 29,4 |  | 53 | HC | Ref |  | 4 | 0 | 2 | 6 |
| Ohyama, 2002 | Yokohama, Japan | Cohort | CA | Retro | 143 (1) | 1162 | 27,8 |  |  | HC | Ref |  | 4 | 0 | 2 | 6 |
| Pappas, 2014 | USA | Cohort | CA | Prosp | 2390 (16) |  | 24,4 | 51 | 75 | HC | Ref | Ref | 4 | 0 | 3 | 7 |
| Park, 2016 | Seoul, Korea | Cohort | ROP | Retro | 201 (1) | 1767 | 32,1 |  |  | CC | NoDes | ICROP | 3 | 0 | 2 | 5 |
| Perrone, 2012 | Siena, Italy | Cohort | CA | Prosp | 92 (1) | 1007 | 26,8 |  |  | HC | Ref | ICROP | 4 | 0 | 3 | 7 |
| Polam, 2005 | New Brunswick, USA | Cohort | CA | Prosp | 177 (1) | 955 | 26,5 | 53 | 74 | HC | Des |  | 4 | 0 | 2 | 6 |
| Rocha, 2006 | Porto, Portugal | Cohort | CA | Retro | 452 (3) | 1499 | 29,4 |  | 65 | HC | Ref | ICROP | 4 | 0 | 3 | 7 |
| Sato, 2011 | Yokohama, Japan | Cohort | CA | Retro | 302 (1) | 1211 | 26,3 | 52 | 61 | HC | Ref | Treat | 4 | 0 | 3 | 7 |
| Schlapbach, 2010 | Zürich, Switzerland | Ca-Co | CA | Retro | 99 (1) | 1244 | 29,1 | 49 | 83 | HC & CC combined | NoDes | NA | 3 | 0 | 2 | 5 |
| Seliga-Siwecka, 2012 | Warsaw, Poland | Cohort | CA | Prosp | 383 (1) | 1338 | 29,2 | 56 | 49 | HC | Ref | Ref | 4 | 0 | 3 | 7 |
| Serenius, 2004 | Sweden | Cohort | ROP | Retro | 140 |  |  |  |  | CC |  | ICROP | 3 | 0 | 3 | 6 |
| Slidsborg, 2016 | Denmark | Cohort | ROP | Retro | 6490 |  |  | 54 |  | CC | NoDes | ICROP | 3 | 0 | 3 | 6 |
| Soraisham, 2009 | Canadian Neonatal Network | Cohort | CA | Prosp | 3094 (24) | 1316 | 28,9 | 53 | 79 | CC | Des | ICROP | 4 | 2 | 3 | 9 |
| Soraisham, 2013 | Regional NICU Southern Alberta, Canada | Cohort | CA | Retro | 384 (1) | 885 | 26,3 | 51 | 86 | HC | Des | NA | 3 | 0 | 2 | 5 |
| Suppiej, 2009 | Padova, Italy | Cohort | CA | Prosp | 104 (1) | 1078 | 28,5 | 46 | 87 | HC | Ref | Ref | 4 | 0 | 3 | 7 |
| Tsiartas, 2013 | Králove, Czech Republic | Cohort | CA | Unclear | 231 (1) | 1975 | 33,0 |  | 56 | HCF | Ref | NA | 4 | 0 | 2 | 6 |
| van Vliet, 2012 | Amsterdam, Netherlands | Cohort | CA | Prosp | 72 (1) | 1117 | 29,0 | 51 | 82 | HC | Des | Ref | 4 | 2 | 3 | 9 |
| Wirbelauer, 2011 | Wuerzburg, Germany | Cohort | CA | Prosp | 71 (1) | 1117 | 0,0 | 52 | 94 | HCF | Ref | Ref | 4 | 0 | 3 | 7 |
| Woo, 2012 | Seoul, Korea | Cohort | ROP | Retro | 246 (1) | 1257 | 29,2 | 57 | 80 | HC & CC separately defined, HCF | Ref | Ref | 4 | 0 | 3 | 7 |

^a^Abbreviations for study design: Ca-Co: case-control study; Perspective, CA: study analyzed ROP as outcome of chorioamnionitis; Perspective, ROP: study analyzed chorioamnionitis as risk factor for ROP; Prosp: prospective; Retro: retrospective;
^b^Chorioamnionitis category: CC: clinical chorioamnionitis; HC: histological chorioamnionitis; HCF: histological chorioamnionitis with funisitis mentioned separately.
^c^Definition of chorioamnionitis: NoDes: no description; Des: clinical or histological description; Ref: defined according to cited article.
^d^Definition of ROP: ICROP: International Classification of Retinopathy of Prematurity; Ref: defined according to cited article; Treat: laser treatment of ROP; NA: no diagnostic criteria mentioned.
^e^Abbreviations for Newcastle-Ottawa Scale: Select: selection; Comp: comparison; Outc: outcome; Tot: total score.
