# Supplementary material for: Chorioamnionitis as a risk factor for retinopathy of prematurity: An updated systematic review and meta-analysis
Source: PLoS One. 2018 Oct 17;13(10):e0205838. doi: 10.1371/journal.pone.0205838 (PMC6192636; doi:10.1371/journal.pone.0205838)
Supplement: S3 Table — Log: logarithm; OR: odds ratio; ROP: retinopathy of prematurity; k: number of studies included; CI: confidence interval. (DOCX) [file pone.0205838.s010.docx]

**S3 Table. Meta-regression of risk of confounding factors and risk of ROP**

| Meta-regression | ROP stage | *k* | Coefficient | 95% CI | Z | *p* |
| --- | --- | --- | --- | --- | --- | --- |
| **Chorioamnionitis type (clinical/histological)** | All ROP | 30 | 0.15 | -0.35 to 0.66 | 0.58 | 0.560 |
|  | Severe ROP | 28 | -0.21 | -0.52 to 0.09 | -1.36 | 0.174 |
| **Antenatal corticosteroids (log OR)** | All ROP | 13 | 0.36 | -0.07 to 0.80 | 1.64 | 0.102 |
|  | Severe ROP | 17 | -0.08 | -0.63 to 0.46 | -0.30 | 0.761 |
| **Cesarean section (log OR)** | All ROP | 10 | -0.10 | -0.85 to 0.66 | -0.25 | 0.801 |
|  | Severe ROP | 16 | -0.21 | -0.43 to 0.02 | -1.83 | 0.067 |
| **Early onset sepsis (log OR)** | All ROP | 7 | 0.65 | 0.23 to 1.07 | 3.03 | 0.003 |
|  | Severe ROP | 11 | 0.23 | -0.46 to 0.42 | -0.07 | 0.946 |
| **Late onset sepsis (log OR)** | All ROP | 8 | -0.13 | -0.80 to 0.54 | -0.39 | 0.695 |
|  | Severe ROP | 12 | 0.49 | -0.05 to 1.03 | 1.76 | 0.078 |
| **Small for gestational age (log OR)** | All ROP | 5 | 0.58 | 0.16 to 1.00 | 2.70 | 0.007 |
|  | Severe ROP | 9 | 0.41 | 0.03 to 0.79 | 2.10 | 0.036 |
| **Premature rupture of membranes (log OR)** | All ROP | 5 | -0.46 | -1.21 to 0.29 | -1.19 | 0.233 |
|  | Severe ROP | 9 | -0.20 | -0.44 to 0.05 | -1.58 | 0.113 |
| **Mortality (log OR)** | All ROP | 11 | 0.30 | -0.24 to 0.85 | 1.10 | 0.273 |
|  | Severe ROP | 13 | 0.74 | 0.37 to 1.11 | 3.95 | <0.001 |

Log: logarithm; OR: odds ratio; ROP: retinopathy of prematurity; *k*: number of studies included; CI: confidence interval.
