# Supplementary material for: Chorioamnionitis as a risk factor for retinopathy of prematurity: An updated systematic review and meta-analysis
Source: PLoS One. 2018 Oct 17;13(10):e0205838. doi: 10.1371/journal.pone.0205838 (PMC6192636; doi:10.1371/journal.pone.0205838)
Supplement: S4 Table — BW: birth weight; GA: gestational age; Histol.: histological; OR: odds ratio; ROP: retinopathy of prematurity. (DOCX) [file pone.0205838.s011.docx]

**S4 Table. Meta-analysis of crude and adjusted risk of all stages ROP.**

| Study or subgroup | | Unadjusted OR (95% CI) | *p* | Adjusted OR  (95% CI) | *p* | Confounders included in analysis |
| --- | --- | --- | --- | --- | --- | --- |
| Clinical | Damman | 4.95 (1.28 to 18.98) | 0.020 | 1.80 (0.32 to 10.02) | 0.502 | GA <29 |
|  | Soraisham 2009 | 1.68 (1.25 to 2.25) | 0.001 | 1.17 (0.62 to 2.53) | 0.538 | GA, BW, vaginal delivery, antenatal steroids, maternal hypertension, Apgar score 5 min, illness severity |
|  | **Clinical** | 2.02 (1.10 to 3.70) | 0.024 | 1.25 (0.62 to 2.53) | 0.538 |  |
| Histol. | Mu | 1.20 (0.58 to 2.47) | 0.621 | 1.53 (0.71 to 3.31) | 0.278 | GA |
|  | Van Vliet | 5.00 (1.07 to 23.30) | 0.040 | 1.59 (0.09 to 27.48) | 0.750 | GA, BW z-score, cesarean delivery |
|  | **Histol.** | 1.82 (0.70 to 4.73) | 0.220 | 1.54 (0.52 to 4.56) | 0.437 |  |
| **Overall** | | 1.73 (1.33 to 2.25) | <0.001 | 1.29 (0.87 to 1.91) | 0.203 |  |

BW: birth weight; GA: gestational age; Histol.: histological; OR: odds ratio; ROP: retinopathy of prematurity.
