# Supplementary material for: Chorioamnionitis as a risk factor for retinopathy of prematurity: An updated systematic review and meta-analysis
Source: PLoS One. 2018 Oct 17;13(10):e0205838. doi: 10.1371/journal.pone.0205838 (PMC6192636; doi:10.1371/journal.pone.0205838)
Supplement: S5 Table — BW: birth weight; GA: gestational age; Histol.: histological; OR: odds ratio; ROP: retinopathy of prematurity. (DOCX) [file pone.0205838.s012.docx]

**S5 Table. Meta-analysis of crude and adjusted risk of severe ROP (stage ≥3).**

| Study or subgroup | | Unadjusted OR (95% CI) | *p* | Adjusted OR  (95% CI) | *p* | Confounders included in analysis |
| --- | --- | --- | --- | --- | --- | --- |
| **Clinical** | Gagliardi | 2.61 (2.08 to 3.27) | <0.001 | 1.48 (1.02 to 2.15) | 0.039 | GA, antenatal steroids, gender, multiple pregnancies, inborn/outborn, mode of delivery |
|  | Garcia-Muñoz | 1.68 (1.29 to 2.20) | <0.001 | 0.88 (0.59 to 1.32) | 0.547 | GA, BW, sex, maternal hypertension, antenatal steroids, maternal antibiotics, multiplicity, type of delivery, necessity of advanced resuscitation, and CRIB 1 |
|  | **Clinical** | 2.11 (1.43 to 3.12) | <0.001 | 1.15 (0.69 to 1.91) | 0.587 |  |
| **Histol.** | Kim | 2.00 (1.07 to 3.74) | 0.029 | 1.19 (0.49 to 2.88) | 0.700 | GA, BW |
|  | Mu | 1.22 (0.45 to 3.29) | 0.695 | 1.33 (0.46 to 3.82) | 0.596 | GA |
|  | **Histol.** | 1.74 (1.03 to 2.95) | 0.040 | 1.25 (0.63 to 2.46) | 0.525 |  |
| **Overall** | | 2.00 (1.46 to 2.74) | <0.001 | 1.17 (0.88 to 1.56) | 0.271 |  |

BW: birth weight; GA: gestational age; Histol.: histological; OR: odds ratio; ROP: retinopathy of prematurity.
